# Navigating workplace uncertainty: a path analysis of perceived overqualification, covert narcissism, workplace alienation, and role ambiguity among nurses

**DOI:** 10.1186/s12912-025-03166-x

**Published:** 2025-05-16

**Authors:** Heba Sobhy Mohamed, Mohammed Adel Abd Elhafeez Elbakry, Ahmed Abdellah Othman, Mohamed Hussein Ramadan Atta, Abeer Moustafa Barakat, Alaa Eldin Moustafa Hamed

**Affiliations:** 1 Nursing Administration, Faculty of Nursing, Zagazig University, Zagazig, Egypt; 2 Nursing Administration, Faculty of Nursing, Sohag University, Sohag, Egypt; 3 Nursing Department College of Applied Medical Sciences, Prince Sattam Bin Abdulaziz University, Wadi Addawasir City, Saudi Arabia; 4 Psychiatric-Mental Health Nursing, Faculty of Nursing, Alexandria University, Alexandria, Egypt; 5 Maternal and Newborn Health Nursing Faculty of Nursing, Helwan University, Cairo, Egypt; 6 Psychiatric and Mental Health Nursing, Faculty of Nursing, Helwan University, Cairo, Egypt

**Keywords:** Perceived overqualification, Covert narcissism, Workplace alienation, Role ambiguity

## Abstract

**Background:**

Perceived overqualification is an emerging challenge in the nursing workforce, influencing job satisfaction, psychological well-being, and overall retention. This study aims to examine the relationships among perceived overqualification, covert narcissism, workplace alienation, and role ambiguity among nurses using path analysis to explore their interactions.

**Methods:**

A cross-sectional study was conducted with 446 nurses from various Egyptian healthcare settings. Data was collected through structured face-to-face interviews using four validated instruments: the Scale of Perceived Overqualification (assessing subjective overqualification), Hypersensitive Narcissism Scale (measuring covert narcissism), Role Ambiguity Scale (evaluating clarity in job responsibilities), Work Alienation Scale (assessing workplace alienation), Additionally, a researcher-developed Demographic Questionnaire was used to collect participants’ background characteristics. Path analysis was conducted using AMOS 26.0 to test direct and indirect relationships among variables, with model fit assessed using standard indices.

**Results:**

Direct effects revealed that perceived overqualification significantly predicted workplace alienation (B = 0.401, 95% CI [0.311, 0.490], *p* <.001), covert narcissism (B = 0.430, 95% CI [0.193, 0.423], *p* <.001), and role ambiguity (B = 0.603, 95% CI [0.150, 0.463], *p* <.001). Additionally, workplace alienation showed positive direct associations with role ambiguity (B = 0.268, 95% CI [0.195, 0.443], *p* <.001) and covert narcissism (B = 0.191, 95% CI [0.293, 0.426], *p* <.001). Mediated effects were also observed: perceived overqualification indirectly influenced workplace alienation via covert narcissism (B = 0.367, 95% CI [0.131, 0.273], *p* <.001) and via role ambiguity (B = 0.228, 95% CI [0.183, 0.513], *p* <.001).

**Conclusion:**

Perceived overqualification significantly contributes to workplace alienation among nurses, with covert narcissism and role ambiguity acting as mediators. To mitigate these effects, healthcare organizations should implement targeted interventions such as structured role clarification, career development programs, and leadership strategies that enhance nurses’ sense of professional fulfillment. These strategies can improve job satisfaction, reduce turnover, and support workforce sustainability in healthcare settings.

**Clinical trial number:**

Not applicable.

## Background

Perceived overqualification negatively affects both individuals and organizations. Feeling overqualified typically reduces job satisfaction, increases turnover intention, and diminishes psychological well-being, leading to disengagement from work [1, 2]. Despite the persistent demand for highly qualified and educated nurses, many hospitals fail to align job roles with nurses’ qualifications, resulting in dissatisfaction and increased workplace stress [3]. Some healthcare institutions provide career advancement opportunities, whereas others fail to recognize nurses’ full potential, leading to frustration and alienation from work. This misalignment is often explained by the Person-Job Fit Theory, which suggests that when employees perceive a mismatch between their abilities and job demands, they experience dissatisfaction and disengagement [4].

According to Maynard and colleagues (2006), overqualification occurs when nurses possess more education, experience, or abilities than what their job requires [5]. It can be categorized into objective and perceived overqualification. Objective overqualification refers to excessive formal education, while perceived overqualification is the subjective feeling of being underutilized [6]. Research suggests that perceived overqualification has a more significant impact on employees’ attitudes and behaviors than objective overqualification [7]. Overqualified nurses often experience reduced organizational commitment and an increased likelihood of developing negative workplace attitudes [8].

Workplace alienation, a key consequence of perceived overqualification, refers to a psychological state in which nurses feel detached from their work environment [9]. The Job Demands-Resources (JD-R) Model suggests that job stressors, such as role ambiguity and perceived overqualification, deplete employees’ emotional resources, leading to alienation and burnout [10]. Prior studies have indicated that overqualified nurses are more likely to experience workplace alienation due to unmet expectations, lack of career growth, and feeling undervalued [3, 11]. These findings highlight the need to address workplace alienation through interventions that improve role clarity and provide professional development opportunities.

Covert narcissism, a less overt form of narcissistic behavior, is characterized by hypersensitivity to criticism, entitlement, and an inflated self-perception [12]. Unlike overt narcissism, which is associated with grandiosity and dominance, covert narcissism is linked to feelings of inferiority, resentment, and increased sensitivity to workplace dynamics [13]. Studies suggest that employees with covert narcissistic traits may struggle with workplace relationships, feel underappreciated, and exhibit defensive behaviors in response to perceived slights [14]. In nursing, covert narcissism may exacerbate the effects of perceived overqualification by intensifying feelings of alienation and dissatisfaction [15].

Role ambiguity is another workplace factor that influences job satisfaction and employee well-being. It occurs when nurses are uncertain about their job responsibilities, expectations, or authority, leading to stress and reduced job performance [16]. Research has shown that role ambiguity is linked to job dissatisfaction, emotional exhaustion, and increased turnover rates among nurses [17]. The JD-R Model further explains that role ambiguity acts as a workplace stressor, draining employees’ psychological resources and increasing workplace alienation [10].

Although perceived overqualification may lead to workplace alienation among nurses, its directional impact on covert narcissism and role ambiguity requires deeper theoretical grounding. Drawing on self-discrepancy theory and the ego-defense mechanism framework, individuals who perceive a mismatch between their abilities and job roles often experience psychological strain, which may manifest as covert narcissism, a defensive response to unmet self-expectations and professional recognition [18–20]. Furthermore, Person-Job Fit Theory posits that a misalignment between qualifications and job demands can increase uncertainty and confusion about roles, leading to heightened role ambiguity [4]. Recent empirical studies support this directionality, showing that overqualified employees often report increased narcissistic sensitivity and uncertainty in job roles due to unmet expectations and unclear professional identity [21–23]. These findings suggest that POQ may initiate psychological and organizational strain responses, mediated by covert narcissism and role ambiguity.

Given these gaps in the literature, this study explores the interplay between perceived overqualification, covert narcissism, role ambiguity, and workplace alienation among nurses. Drawing on organizational and psychological theories, it examines how these factors contribute to workplace experiences and professional identity within nursing. By analyzing these relationships, the study advances theoretical understanding of workforce dynamics while offering practical insights for improving job satisfaction, reducing turnover, and strengthening organizational commitment [18]. It extends existing frameworks by integrating covert narcissism and role ambiguity as key psychological factors influencing workplace alienation. The findings provide empirical support for targeted strategies that optimize talent utilization, enhance nurse engagement, and inform evidence-based policies for retaining overqualified nurses and fostering a sustainable healthcare workforce. To address this gap, the study proposes the following hypotheses:

- **H1**: POQ is positively and significantly related to work alienation among nurses.
- **H2**: POQ is positively and significantly related to covert narcissism among nurses.
- **H3**: POQ is positively and significantly related to role ambiguity among nurses.
- **H4**: Role ambiguity is positively correlated with work alienation among nurses.
- **H5**: Covert narcissism is positively correlated with work alienation among nurses.
- **H6**: Covert narcissism significantly mediates the relationship between POQ and work alienation among nurses.
- **H7**: Role ambiguity significantly mediates the relationship between POQ and work alienation among nurses.

## Methods

### Aim of the study

The present study aims to explore perceived over-qualification, covert Narcissism, workplace alienation, and role ambiguity among nurses.

### Setting and study design

A cross-sectional study was conducted with 446 nurses from various Egyptian healthcare settings. Between April and October 2024, data were collected through structured face-to-face interviews using five validated instruments: the Perceived Overqualification Scale (assessing subjective overqualification), Hypersensitive Narcissism Scale (measuring covert narcissism), Role Ambiguity Scale (evaluating clarity in job responsibilities), Work Alienation Scale (assessing workplace alienation), and a Demographic Questionnaire. A stratified random cluster sampling technique was employed to ensure representation across different hospital units, regions, and professional backgrounds. This approach was specifically chosen to enhance methodological transparency and increase the generalizability of the study findings within the context of Egyptian nursing settings. First, hospitals were categorized based on size and type (public, private, and university-affiliated). Within each category, clusters were formed based on hospital departments (e.g., medical, surgical, intensive care, etc.), ensuring diversity in work settings. The use of stratification ensured that various subgroups of nurses, such as those working in different departments and regions, were adequately represented. Additionally, hospitals were selected from different geographic regions, including urban and rural areas, to enhance representativeness. Nurses were then randomly selected from these clusters, maintaining proportional representation to reflect the broader nursing workforce distribution, thus increasing the external validity of the findings in relation to the entire nursing population in Egypt.

A total of 600 nurses were invited to participate in the study, of whom 446 completed the survey, yielding a response rate of 74.3%. This response rate suggests adequate representation of the target population, minimizing nonresponse bias. Path analysis was conducted using AMOS 26.0 to test direct and indirect relationships among variables, with model fit assessed using standard indices. Given the cross-sectional design, causal inferences cannot be drawn. The term “mediating” or “indirect” effects in this study refer to statistical associations rather than definitive causal relationships.

#### Sampling and participants

A sample of 446 nurses across diverse regions, including Upper Egypt, Eastern Egypt, Western Egypt, the Delta area, and the Center, were employed. The G*Power (3.1.9.7) program was employed to determine the sample size required for a study, explicitly using calculations based on F tests in the context of linear multiple regression with a fixed model and the R² deviation from zero. The required sample size was determined based on an assumed medium effect size (f² = 0.15), an alpha level of 0.05, and a power of 0.80, ensuring adequate sensitivity for detecting relationships among study variables. A total of *446* nurses, categorized into three age groups: <25 years, 25–40 years, and > 40 years, with various educational backgrounds, including a Diploma Nurse, Technical Institute of Nursing, bachelor’s degree in nursing, and postgraduate nursing education, were recruited. These nurses are currently working in different hospital departments.

### Instruments for data collection

Five instruments were utilized to collect data for this study, including four established and validated instruments, which are publicly available, free to use, and do not require permission for use, as well as a researcher-developed sociodemographic questionnaire. These instruments are described as follows:

### *Tool one*: nurses’ sociodemographic characteristics questionnaire

Created by researchers in their native language (Arabic), this section provides a comprehensive list of the various personal characteristics of nurses, including age, gender, education level, job position, residence, marital status, years of experience, monthly income, department, and types of hospitals. Each item was carefully chosen to paint a complete picture of the diverse demographic and professional attributes of the participating nurses. This meticulous approach to data collection was intended to ensure that the information gathered was both high-quality and relevant, significantly enhancing the understanding of the research context and facilitating more rigorous analyses in future studies.

### 2nd instrument: perceived overqualification (POQ) scale

developed by Maynard et al., (2006) [24]. It is a validated tool used to assess individuals’ perceptions of possessing qualifications that exceed the requirements of their current job roles. The scale consists of nine items, each rated on a 7-point Likert scale ranging from 1 (Strongly Disagree) to 7 (Strongly Agree). A composite score is calculated by summing the responses to all nine items and dividing by nine, resulting in a mean score that represents the overall perception of overqualification. Higher scores indicate a stronger perception of being overqualified, while lower scores suggest a closer alignment between the individual’s qualifications and job requirements. The SPOQ has demonstrated high internal consistency (Cronbach’s alpha = 0.89) and has been widely used to explore the effects of overqualification on workplace attitudes and behaviors. Lee and colleagues reported good internal consistency reliability for this tool, with a Cronbach’s alpha coefficient of 0.90 [25]. Also, other studies conducted by Johnson and colleagues (2002) and Lu and Colleagues (2023) used the same scale among nurses and reported a good Cronbach’s alpha coefficient (Cronbach’s α = 0.78 and 0.848, respectively). In this study, the internal consistency coefficient, Cronbach’s alpha, was 0.821, reflecting high reliability.

### 3rd instrument: hypersensitive narcissism scale (HSNS)

The Hypersensitive Narcissism Scale (HSNS), developed by Hendin and Cheek (1997), is a self-report measure designed to measure individuals’ level of covert narcissism. It consists of ten items, with responses evaluated on a 5-point Likert scale ranging from 1 (Very uncharacteristic or untrue, strongly disagree) to 5 (Very characteristic or true, strongly agree). The composite score for this scale is derived by summing the responses to all ten items, resulting in a total score ranging from 10 (low covert narcissism) to 50 (high covert narcissism). Higher total scores indicate higher levels of covert narcissism. For consistency in interpretation, a higher score represents a greater degree of personal covert narcissism. Moon and Morais (2023) reported good internal consistency reliability for this tool, with a Cronbach’s alpha coefficient of 0.789 [14]. Also, Lachowicz-Tabaczek and colleagues (2021) used the same scale and reported a good Cronbach’s alpha coefficient (Cronbach’s α = 0.65) [14]. In this study, the internal consistency coefficient, Cronbach’s alpha, was 0.792, indicating acceptable reliability.

### 4th instrument: work alienation scale

The Multidimensional scale of work alienation was developed by Mottaz (1981) [26] to assess workplace alienation as experienced by nurses. It consists of 21 items, covering three dimensions: self-estrangements (7 items), with a sample item, “I do not feel a sense of accomplishment in the type of work I do.”, and powerlessness (7 items) with a sample item “I am not able to make changes regarding my job activities,” and meaninglessness (7items) with sample item was “Sometimes I am not sure I completely understand the purpose of what I’m doing.” The responses are assessed on a 5-point Likert scale that ranges from 1 (strongly disagree) to 5 (strongly agree), with higher scores indicating higher levels of work alienation. A composite score was calculated by summing the responses to all 21 items and dividing them by 21, resulting in a mean score representing overall work alienation. Higher mean scores indicate higher levels of work alienation. The range of the scale is 1 to 5, where higher scores indicate higher work alienation. Abd El-Monem and colleagues (2023) reported good internal consistency reliability for this tool, with a Cronbach’s alpha coefficient of 0.887 [27]. Another study performed by Moahmed and Abou Shreen (2022) used the same scale among nurses and reported a good Cronbach’s alpha coefficient (Cronbach’s α = 0.76) [28]. In our study, the internal consistency coefficient Cronbach’s alpha value of this scale was 0.835, indicating acceptable reliability.

### 5th instrument: role ambiguity scale

The Role Ambiguity scale was developed by Rizzo, House, and Lirtzman (1970) [29]. It’s used to measure role ambiguity among nurses. Responses are assessed on a 7-point Likert scale that ranges from 7 (strongly agree) to 1 (strongly disagree), with higher scores associated with higher levels of role ambiguity. A composite score was calculated by summing the responses to all items and dividing by the number of items, resulting in a mean score representing overall role ambiguity. Higher mean scores indicate higher levels of role ambiguity. The range of the scale is 1 to 7, where higher scores indicate higher role ambiguity. This scale has been widely used in previous years, as demonstrated by Smith and colleagues (1993), who reported good internal consistency reliability for this tool, with Cronbach’s alpha coefficients ranging from 0.73 to 0.80 for the items [30]. This established reliability underscores the scale’s effectiveness in measuring the intended construct. Alyahya and colleagues (2021) used the same scale among nurses and reported that the scale displayed high levels of validity, internal consistency, and test-retest reliability [31]. In this study, the internal consistency coefficient, Cronbach’s alpha, was 0.902, indicating high reliability.

### Procedure

Before the commencement of the primary research study, a preliminary investigation, referred to as a pilot study, was conducted on a randomly selected cohort of 45 nurses, constituting 10% of the total nurses under examination. The primary objectives of this pilot study were to assess the clarity, applicability, and practicality of the study instruments, gauge the time required for participants to complete the instruments, and identify any potential challenges anticipated during data collection. The results from the pilot study indicated that the instruments were largely clear and effective. However, a few minor issues were identified: some participants required additional time to complete the questionnaires, which prompted slight revisions in the item structure to improve clarity. The average interview time was found to be approximately 20 min, which was deemed appropriate. Additionally, one item on workplace alienation was noted as slightly confusing by some participants, leading to a revision for clarity. Notably, participants involved in the pilot study were deliberately excluded from the overall sample of the research to ensure result consistency.

Since all standardized instruments were originally developed in English, two translation methods were employed to ensure linguistic and conceptual equivalence in Arabic: the committee-based approach and the forward–backward translation method [32, 33]. The committee approach involved a multidisciplinary panel of bilingual nursing researchers, psychiatric nursing experts, and a linguistic specialist who collaboratively translated and adapted the scales to ensure cultural appropriateness, conceptual accuracy, and clarity. Additionally, a rigorous forward–backward translation process was conducted, where two independent bilingual nursing researchers first translated the scales into Arabic. A third expert in psychiatric nursing and mental health reviewed the translations to ensure clarity and cultural relevance. Subsequently, a separate bilingual translator, unfamiliar with the original instruments, performed a back-translation into English. The original and back-translated versions were compared, and discrepancies were discussed and resolved by a panel of experts to ensure both semantic and conceptual accuracy.

To further ensure clarity and comprehension, the Arabic versions underwent pilot testing on a group of 45 nurses (excluded from the final sample). Based on participant feedback, minor wording adjustments were made to enhance readability while preserving the original meaning of each item. Some linguistic and conceptual issues identified during the pilot testing included unclear phrasing in certain items. These were resolved through discussions with bilingual nursing researchers and a linguistic expert, leading to revisions that ensured the clarity of the instruments while retaining their original intent. Additionally, the content validity of the translated instruments was evaluated by a panel of five experts in nursing research and psychology, confirming their suitability for Arabic-speaking populations.

During the data collection phase, we met the participant nurses and conducted face-to-face interviews with them in various hospitals; the research objectives were explained to the nurses, and their ability to participate was assessed. Upon obtaining their written consent, we scheduled and conducted in-person interviews at times suitable for the nurses. These interviews were arranged in strategically positioned areas in the hospitals to guarantee privacy and minimal distraction for both the nurses and the researcher. To minimize social desirability bias, participants were reassured that their responses would remain anonymous and confidential, emphasizing that no identifying information would be linked to their answers. Additionally, they were informed that their participation was voluntary and that there were no right or wrong responses, encouraging them to answer as honestly as possible. To ensure standardization and minimize interviewer bias, all interviews followed a structured interview guide that outlined specific question phrasing, sequencing, and prompts.

Additionally, the researchers underwent training on interview techniques to enhance consistency, neutrality, and active listening. Guidelines were established to maintain uniformity in the way questions were asked and responses were recorded. Moreover, sensitive constructs such as covert narcissism and workplace alienation were measured using indirect questioning techniques and neutral wording to reduce potential response distortion. For instance, instead of directly asking about narcissistic tendencies, the instrument included behaviorally anchored items that assess personality traits in everyday work scenarios, making the questions feel less judgmental or intrusive.

The researchers followed a structured questionnaire to gather responses, ensuring consistency and accuracy in data collection. The survey was comprised of two parts: the first part was the informed consent, which explained the study’s aim, confidentiality guarantees, and the ability to withdraw at any time. This informed consent form was included in one document with the research tool. Participants had to agree to the terms before continuing with the questionnaire, ensuring they fully understood the study before proceeding. The second part included the research instrument. In this phase, efforts were made to create a comfortable and supportive environment, encouraging honest and comprehensive responses from the participants.

### Ethical consideration

Approval from the Research Ethics Committee (REC) of the College of Nursing, Zagazig University, Egypt (Approval ID: ZU.Nur.REC#:153) was obtained in March 2024. The study’s objective was communicated to the participants involved, ensuring that all data collected would be utilized solely for research reasons. Also, each participant was notified of their right to decline participation or withdraw from the study before finishing the study materials without facing negative repercussions. The study sought informed written consent from nurses who agreed to participate. To maintain confidentiality and data security, anonymized responses, ensuring that data was used exclusively for research purposes. Access to this data was restricted to the research team only. All procedures of the study were conducted by the ethical guidelines established by Helsinki and its later amendments, ensuring that the rights and welfare of all participants were protected throughout the research process.

### Data analysis

Data analysis was performed using SPSS 26.0 (IBM Inc., Chicago, IL, USA) to examine the survey responses from the 446 recruited nurses. Descriptive statistics, such as frequencies (percentages) and mean ± standard deviations (SD), were utilized to summarize both the general characteristics of the participants and the scores obtained on various scales. Correlations between perceived over-qualification, covert narcissism, workplace alienation, and role ambiguity among nurses were evaluated using Pearson’s correlation analysis. ANOVA and t-test were utilized to examine the relation between study variables and personal data. The mediating function of was tested using JASP 0.14.1.0. Statistical significance was interpreted at the level of *P* ≤.05.

To ensure the integrity of the study results, missing data were managed with pairwise deletion. This method excluded participants with missing data for specific analyses, avoiding imputation and maintaining the robustness of the analysis. Only complete cases, with no missing data on key variables, were included in the path analysis, preventing bias and ensuring valid results. This approach upheld the quality and reliability of the findings, in line with best practices for cross-sectional data analysis.

Before conducting path analysis, we examined the data for normality and the presence of outliers. Normality was assessed using visual methods, such as histograms and Q-Q plots, along with statistical tests, including the Shapiro-Wilk test. Outliers were identified through standardized z-scores, and any values beyond the accepted threshold (e.g., ± 3.29) were considered for further investigation. Any extreme outliers that could unduly influence the model were either adjusted or excluded as per best practices in structural equation modeling. This ensured that the data used for path analysis were suitable and reliable for statistical modeling.

To investigate the relationships between perceived overqualification, covert narcissism, workplace alienation, role ambiguity, and demographic characteristics among nurses, a path analysis model was developed and tested using Amos 26.0 (Amos Development Corp, Meadville, PA, United States). The path analysis explored the direct and indirect associations among these variables, including demographic factors, to understand the impact on workplace dynamics. Model fit was assessed using standard fit indices. The results indicate an acceptable model fit: χ²/df = 2.89, RMSEA = 0.062 (90% CI: 0.048–0.075), CFI = 0.928, TLI = 0.912, and SRMR = 0.059. These values align with established criteria for model adequacy, supporting the hypothesized relationships within the study.

## Result

Table 1 shows that the majority of participants were female nurses aged 25 to < 35 years, holding a diploma from a Nursing Technical Institute, and residing in rural areas. Significant associations were observed between perceived overqualification and age, educational level, and years of experience, with higher scores among nurses aged 35 to ≤ 40 years, those with postgraduate education, and those with ≥ 10 years of experience. Role ambiguity was notably higher among younger nurses, diploma holders, and those with less experience. Additionally, workplace alienation was significantly linked to educational level, with bachelor’s degree holders reporting the highest scores. In contrast, covert narcissism showed no significant demographic variations. See Table 1 for detailed statistics. Insert Table 1.

**Table 1:** Relationship between total study variables and studied Nurse’s characteristics (*n* = 446)

Personal characteristics	*N* (%)	Perceived overqualification			Covert narcissism			Role ambiguity			Workplace alienation		
		M (SD)	t/F (*P*)	df	M (SD)	t/F (*P*)	df	M (SD)	t/F (*P*)	df	M (SD)	t/F (*P*)	df
Age /F-test													
< 25	78(17.5)	2.102 ± 0.797	0.752 (< 0.01)**	2	2.910 ± 0.9827	0.564 (0.570)	2	3.20 ± 1.290	0.585 (< 0.01)**	2	3.115 ± 1.031	1.336 (0.264)	2
25-<35	319(71.5)	2.256 ± 0.9319			3.05 ± 1.1031			3.094 ± 1.275			3.250 ± 1.1518		
35 ≥ 40	49(11.0)	2.285 ± 1.007			3.000 ± 1.274			2.961 ± 1.273			3.000 ± 1.020		
Gender/ F-test													
Male	83(18.6)	2.220 ± 0.8722	1.806 (0.072)	444	3.096 ± 1.054	0.657 (0.498)	444	2.915 ± 1.317	1.326 (0.199)	444	3.120 ± 0.942	0.713 (0.476)	444
Female	363(81.4)	2.433 ± 0.9921			3.008 ± 1.113			3.121 ± 1.264			3.217 ± 1.1560		
Education level / F-test													
Diploma / Nursing Technical Institute	324(72.6)	1.416 ± 0.5149	4.704 (< 0.001)**	2	3.006 ± 1.1238	1.978 (0.140)	2	3.249 ± 1.2920	0.442 (< 0.01)**	2	3.148 ± 1.0972	3.675 (0.026)	2
Bachelor’s degree in nursing	110(24.7)	2.281 ± 0.9299			3.136 ± 1.035			3.181 ± 1.2792			3.40 ± 1.183		
Post graduated education	12(2.7)	2.484 ± 0.9889			2.500 ± 1.000			3.083 ± 0.6685			2.666 ± 0.7785		
Place of Residence / t-test													
Urban	154(34.5)	2.181 ± 0.966	1.234 (0.218)	444	3.02 ± 1.143	0.018 (0.985)	444	2.961 ± 1.277	1.468 (0.143)	444	3.181 ± 1.1228	243 (0.808)	444
Rural	292(65.5)	2.301 ± 0.976			3.024 ± 1.0822			3.147 ± 1.271			3.208 ± 1.118		
Marital status / t-test													
Unmarried	359(80.5)	2.259 ± 0.9614	046 (0.964)	444	3.022 ± 1.1058	0.093 (0.926)	444	3.039 ± 1.3132	1.481 (0.139)	444	3.195 ± 1.126	0.175 (0.861)	444
Married	87(19.5)	2.264 ± 1.0282			3.034 ± 1.093			3.264 ± 1.093			3.218 ± 1.093		
Years of experience/ F-test													
1 < 5	139(31.2)	2.050 ± 0.686	1.373 (< 0.01)**	2	3.007 ± 1.032	0.318 (0.728)	2	3.223 ± 1.1423	2.390 (< 0.01)**	2	3.23 ± 1.0022	0.549 (0.578)	2
5 < 10	287(64.3)	2.179 ± 0.9188			3.045 ± 1.1194			3.048 ± 1.3133			3.202 ± 1.1830		
10≥	20(4.5)	2.313 ± 1.0135			2.850 ± 1.3484			2.600 ± 1.500			2.950 ± 0.9445		
Perception Monthly Income/ t–test													
Enough	243(54.5)	2.181 ± 0.966	1.461 (0.352)	444	2.910 ± 0.9827	0.872 (0.054)	444	2.915 ± 1.317	0.343 (0.214)	444	3.115 ± 1.031	1.394 (0.563)	444
Not enough	203(45.5)	2.301 ± 0.976			3.05 ± 1.1031			3.121 ± 1.264			3.250 ± 1.1518		
*M (SD)* denotes Mean (Standard Deviation); *t/F* = independent t-test/one-way ANOVA; *df* = degrees of freedom; *p* = significance level; * p <.05; ** p <.01.													

As shown in Table 2, perceived overqualification, covert narcissism, role ambiguity, and workplace alienation were all positively correlated. The strongest association was between perceived overqualification and workplace alienation, suggesting that nurses who feel overqualified may be more prone to workplace disengagement. These findings highlight the need for interventions that address overqualification-related stressors to enhance nurse well-being and patient care. All study scales demonstrated good internal consistency, with Cronbach’s alpha values exceeding 0.79. See Table 2 for detailed statistics. Insert Table 2.

**Table 2:** Descriptive analysis & Correlation between study variables among participants (*n* = 446)

Variables	Mean ± SD	α	1	2	3	4
Perceived Overqualification (1)	2.250 ± 0.978	0.821	1	^*^		
Covert Narcissism (2)	3.030 ± 1.105	0.792	0.376^**^ (< 0.001)	1		
Role Ambiguity (3)	3.873 ± 1.281	0.902	0.453^**^(< 0.001)	0.462^**^(< 0.001)	1	
Workplace Alienation (4)	3.990 ± 1.125	0.835	0.562^**^(< 0.001)	0.466^**^(< 0.001)	0.579^**^(< 0.001)	1

M ± SD = Mean ± Standard Deviation; α = Cronbach’s Alpha; r = Pearson correlation coefficient; * *p* <.05; ** *p* <.01

Table 3 presents the direct, indirect, and total effects of perceived overqualification on workplace alienation among nurses. The findings reveal that perceived overqualification has both direct and indirect influences on workplace alienation. The strongest direct effect was observed between perceived overqualification and role ambiguity, while workplace alienation was significantly influenced by role ambiguity and covert narcissism. Additionally, the indirect effects suggest that perceived overqualification contributes to workplace alienation through covert narcissism and role ambiguity, highlighting the mediating roles of these variables. Insert Table 3.

**Table 3:** Mediating effect of role ambiguity and Covert narcissism between perceived overqualification and workplace alienation (*n* = 446)

Direct effect	(B)	Confidence intervals (CI) 95%	Std.error	t	*p*
Perceived Overqualification → Workplace Alienation	0.401	(0.311–0.490)	0.043	8.787	< 0.001^**^
Perceived Overqualification → Covert Narcissism	0.430	(0.193–0.423)	0.032	5.260	< 0.001^**^
Perceived Overqualification → Role Ambiguity	0.603	(0.150-0.463)	0.046	7.355	< 0.001^**^
Role Ambiguity → Workplace Alienation	0.268	(0.195–0.443)	0.027	6.960	< 0.001^**^
Covert Narcissism → Workplace Alienation	0.191	(0.293–0.426)	0.018	5.440	< 0.001^**^
Indirect effect					
Perceived Overqualification → Covert Narcissism → Workplace Alienation	0.367	(0.131–0.273)	0.051	4.968	< 0.001^**^
Perceived Overqualification → Role Ambiguity → Workplace Alienation	0.228	(0.183–0.513)	0.035	6.215	< 0.001^**^
Total effect					
Perceived Overqualification → Workplace Alienation	0.475	(0.281–0.387)	0.026	4.554	< 0.001^**^

*B* = unstandardized regression coefficient; *CI* = confidence interval; *Std. Error* = standard error of *B*; *t* = t-value; *p* = significance level; → indicates mediation path; * p <.05; ** p <.01

**Fig. 1:**
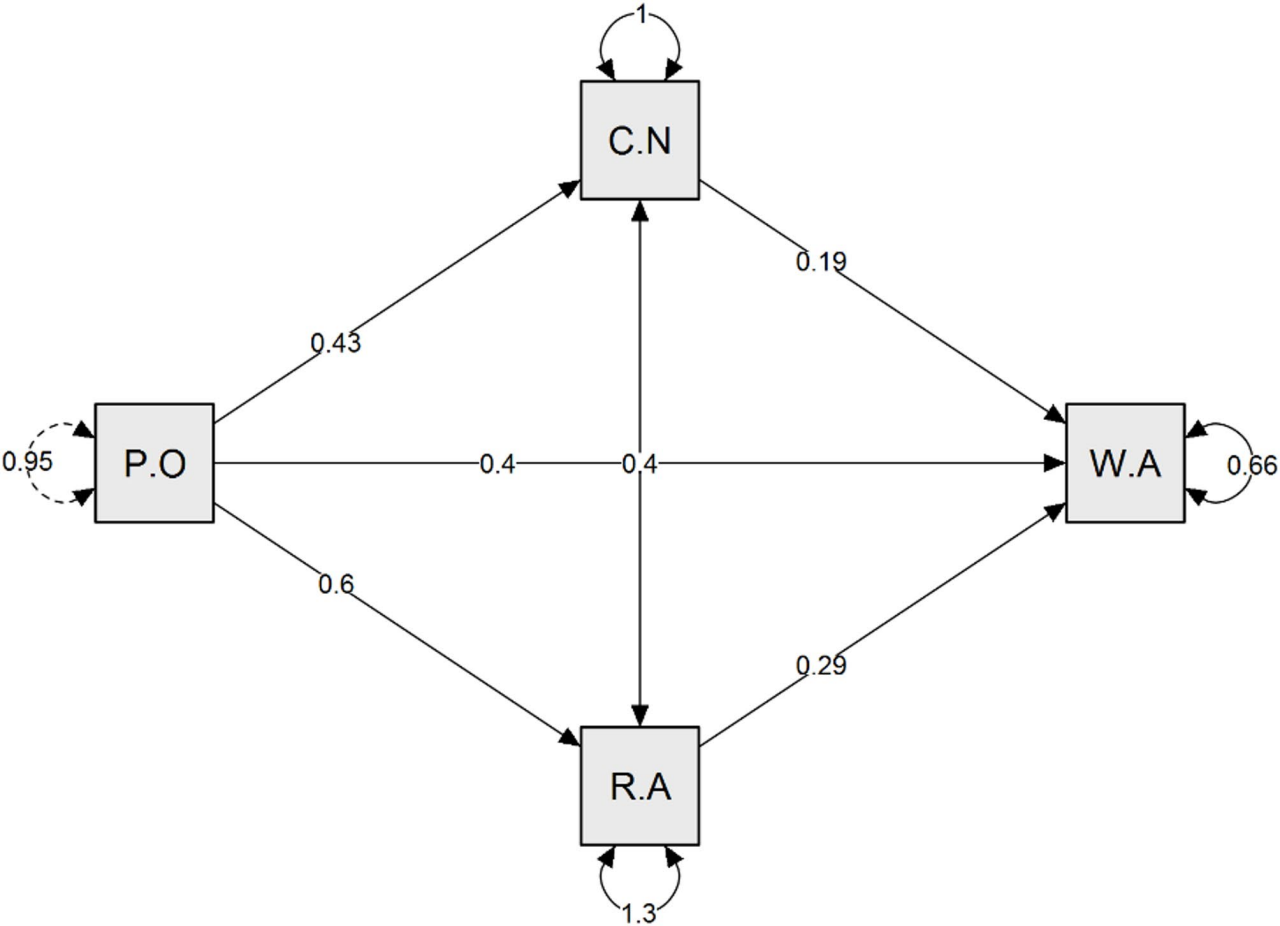
A path mediation analysis of role ambiguity and covert narcissism between perceived overqualification and workplace alienation (*n* = 446). **P O**, Perceived Overqualification; **CN**, Covert Narcissism; **RA**, Role Ambiguity; **WA**, Workplace alienation

Figure 1 illustrates the path analysis model, demonstrating the relationships among perceived overqualification, covert narcissism, role ambiguity, and workplace alienation. The model underscores the interconnected nature of these factors, emphasizing how perceived overqualification can shape workplace alienation through multiple pathways. These findings highlight the importance of addressing perceived overqualification and its psychological consequences to foster a more supportive work environment for nurses. Insert Fig. 1.

## Discussion

The result found a positive correlation between perceived overqualification and covert narcissism among nurses, which is similar to findings by Wiegand (2023) and Matherne and colleagues (2019) [18, 34]. Individuals who perceive themselves as overqualified may experience frustration or dissatisfaction in their current roles, feeling their skills and abilities are underutilized or unrecognized. This sense of unfulfillment may associate with covert narcissistic tendencies, such as an inflated sense of entitlement or self-importance, expressed subtly through passive-aggressive behavior, withdrawal, or resentment toward colleagues [35, 36]. However, it is important to acknowledge that this study’s cross-sectional design precludes any definitive causal inferences. The associations observed should be interpreted as correlations rather than causal relationships.

Additionally, overqualified nurses may struggle with self-esteem, leading to a need for validation and admiration, core traits of covert narcissism [37]. The mismatch between their perceived capabilities and job responsibilities can foster feelings of inadequacy or superiority, further associating with narcissistic traits [38]. Furthermore, the cross-sectional nature of the study means that we cannot definitively assert that perceived overqualification leads to covert narcissism. Future longitudinal studies would be essential to examine the directionality of this relationship. Moreover, the healthcare environment, with its hierarchical structure, can exacerbate these feelings, as overqualified nurses may feel trapped in roles that do not allow for growth, intensifying their covert narcissistic tendencies as a defense mechanism against feelings of professional stagnation [39].

Our findings support the positive interaction between perceived overqualification and role ambiguity, which can be matched with Khassawneh and colleagues’ (2022) results, showing that perceived overqualification had a stronger and more negative association with employee job demands and satisfaction [40]. This relationship can create significant challenges in healthcare settings, where, for example, a nurse with extensive experience and advanced certifications may feel overqualified if assigned to routine tasks that do not require their full expertise. The cross-sectional design of our study suggests that while overqualified nurses may report feelings of dissatisfaction, we cannot conclusively determine whether role ambiguity is a direct cause of this dissatisfaction. Comparing our results with longitudinal research could provide insight into how these relationships evolve. Specifically, Guo and colleagues (2022) support a positive relationship between perceived overqualification, organizational commitment, and work passion of nurses [41].

Additionally, role ambiguity could increase perceived overqualification in cases where experienced nurses are placed in units where roles are loosely defined, such as in new or understaffed wards. These results are consistent with those of Guo and colleagues (2022), who found that role ambiguity increases nurses’ perceptions of overqualification, particularly in environments with unclear job expectations [8]. Without clear guidance on protocols or expectations, these nurses may feel their expertise is underutilized, further heightening their sense of being overqualified for the position [42]. This could lead to decreased job satisfaction, disengagement from patient care, and a higher likelihood of seeking employment elsewhere. For healthcare systems, this interaction can negatively impact nurses’ well-being and patient care quality [17]. However, since this is a cross-sectional study, we can only infer that role ambiguity and overqualification are related, and not definitively claim that one causes the other. Longitudinal studies are required to examine this dynamic over time.

Interestingly, the positive statistical correlation found between covert narcissism and role ambiguity is a novel relationship. This study contributes new insights into the potential psychological factors underlying role ambiguity in the nursing profession. Covert narcissists often exhibit feelings of insecurity, hypersensitivity to criticism, and a need for validation, but they conceal these traits behind a facade of humility or introversion [43]. In workplace or group settings where roles are unclear, individuals with covert narcissism may experience heightened anxiety and confusion as they struggle to align their self-perception with external expectations. While our study found a significant correlation between these variables, it is critical to note that the cross-sectional nature limits our ability to ascertain whether role ambiguity leads to covert narcissism, or if covert narcissism amplifies the experience of role ambiguity. Future research, particularly longitudinal designs, could offer greater insight into these dynamics.

The statistically significant correlation between role ambiguity and workplace alienation among nurses underscores a critical issue within the nursing profession. Similarly, Zhang et al., 2023 and Tang et al. 2024 found a positive correlation between role ambiguity and workplace alienation among nurses [17, 22]. This strong positive relationship indicates that as nurses encounter increasing levels of role ambiguity marked by unclear expectations and responsibilities, they are more likely to experience feelings of alienation in their work environment. This finding highlights the importance of clear role definitions within healthcare teams, as ambiguity can hinder nurses’ sense of belonging and satisfaction, ultimately impacting patient care as role ambiguity can lead to confusion and frustration as nurses struggle to navigate their roles without clear guidance [23]. This lack of clarity may foster a sense of disconnect from both colleagues and the organizational mission, ultimately resulting in emotional withdrawal and diminished job satisfaction. In a field where effective communication and teamwork are paramount, the experience of alienation can further compromise the quality of patient care [17]. These findings highlight the importance of establishing clear roles and expectations within healthcare settings, as doing so may enhance nurses’ job satisfaction and improve overall workplace cohesion and patient outcomes. Addressing role ambiguity is essential to fostering a supportive work environment that promotes nurses’ well-being and the quality of care they provide.

The mediating effect of role ambiguity and covert narcissism in the relationship between perceived overqualification and workplace alienation was also identified in our study. While this mediation model adds a new layer to the literature, caution is necessary when interpreting these findings, as the cross-sectional data limits our ability to establish causal mechanisms. Prior studies, such as those by Judeh (2011) and Moon and Morais (2023), support the idea that role ambiguity can mediate the relationship between socialization and organizational [14, 44]. Similarly, Moon and Morais (2023) found that covert narcissism had an indirect effect on workplace incivility via perceived norms for respect. Our study extends these findings by demonstrating how these factors might mediate workplace alienation specifically among nurses.

Nurses who feel overqualified for their positions may experience role ambiguity as their skills and expectations do not align with their job responsibilities. This confusion can lead to inadequacy and alienation from their work environment as they struggle to find meaning in their roles [45]. Overqualified nurses may feel detached from their colleagues, particularly if they perceive that their skills are not being utilized effectively. This disengagement can be compounded by role ambiguity, as unclear expectations hinder collaboration and communication, creating feelings of alienation.

Finally, our findings suggest that nurses with higher levels of education, such as those holding a bachelor’s degree or postgraduate qualifications, report greater levels of perceived overqualification, role ambiguity, and workplace alienation. This pattern may stem from the misalignment between their advanced skills and available job opportunities, leading to frustration and disengagement. Highly educated nurses may also have higher expectations for career advancement and professional recognition, which, if unmet, can contribute to feelings of alienation [45]. Additionally, hierarchical workplace structures and limited decision-making autonomy in nursing roles may intensify these effects, particularly for those with aspirations for leadership or specialized practice. These results support our hypothesis regarding the mediating effects of role ambiguity and covert narcissism.

While these findings are significant, they must be interpreted with caution due to the limitations of the cross-sectional study design. Longitudinal studies are needed to examine how these relationships evolve over time and across different nurse populations. In conclusion, our study provides valuable insights into the relationships between perceived overqualification, covert narcissism, role ambiguity, and workplace alienation among nurses. However, the cross-sectional nature of the study limits the ability to infer causality. Future research, particularly longitudinal studies, is crucial for advancing our understanding of these dynamics and their implications for nurse well-being and healthcare outcomes.

## Conclusion

This study underscores perceived overqualification (POQ) as a significant factor contributing to workplace alienation among nurses in Egyptian healthcare settings, with covert narcissism and role ambiguity identified as key mediators in this relationship. The findings demonstrate that elevated levels of POQ, particularly among experienced and highly educated nurses, are strongly correlated with feelings of alienation. Given the specific challenges within Egyptian healthcare systems, such as resource constraints and hierarchical structures, addressing these dynamics through strategies such as clarifying roles, providing career development opportunities, and offering targeted mental health support can help alleviate alienation, improve job satisfaction, and foster a more engaged nursing workforce. Such interventions would be particularly effective in enhancing the work environment, considering the unique cultural and organizational dynamics in Egypt.

### Implication

The findings of this study hold significant implications for healthcare management and retention strategies in Egypt. Reducing workplace alienation among nurses may require tailored interventions for those who perceive themselves as overqualified. These interventions could include opportunities for career advancement through specialized training programs and the creation of advanced roles that align with nurses’ qualifications. Additionally, involving nurses more actively in decision-making processes and promoting role clarity, particularly for less experienced nurses in Egypt’s evolving healthcare landscape, are critical components in improving job satisfaction. Providing mental health support to address covert narcissistic tendencies, alongside cultural considerations of respect and hierarchy, will be important in mitigating the negative effects of perceived overqualification. By better aligning nurses’ qualifications with their responsibilities, Egyptian healthcare organizations can cultivate a more engaged, satisfied, and committed workforce, which, in turn, will contribute to improved organizational effectiveness and better patient care outcomes.

### Limitation of the study

While the current study provides valuable insights into the relationship between perceived overqualification, covert narcissism, role ambiguity, and workplace alienation among nurses, several limitations must be acknowledged. The cross-sectional design limits the ability to draw causal conclusions, as associations between variables were observed without determining cause and effect. Additionally, the study’s sample was specific to one nursing population, which may restrict the generalizability of the findings to other healthcare settings or professions. The reliance on self-report measures introduces potential response bias, suggesting that future research could benefit from incorporating multi-method approaches. Lastly, key contextual factors, such as organizational culture and leadership styles, were not considered and could offer further insights into workplace dynamics. Despite these limitations, the study contributes significantly to the understanding of these complex relationships and suggests avenues for future research and interventions.

## Data Availability

The corresponding author can provide the datasets used and/or analyzed for this study upon reasonable request.

## Abbreviations

HSNS: Hypersensitive Narcissism Scale
REC: Research Ethics Committee
SPOQ: Scale of Perceived Over Qualification

## References

[1] Erdogan B, Bauer TN. Overqualification at work: A review and synthesis of the literature. Annu Rev Organ Psychol Organ Behav. 2021;8:259–83.

[2] Liu P, Mu Y, Li X. How does perceived overqualification beget workplace incivility? A moderated mediation model based on Kahn’s framework. J Bus Res [Internet]. 2025;186(610):114961. Available from: 10.1016/j.jbusres.2024.114961

[3] Xie Z, Chen Z, Wang W, Pu J, Li G, Zhuang J et al. The effect of career compromise on nurses’ turnover intention: the mediating role of job satisfaction. BMC Nurs. 2024;23(1). 10.1186/s12912-024-02346-5 PMC11441125 39343914

[4] Kristof AL. Person-organization fit: an integrative review of its conceptualizations, measurement, and implications. Pers Psychol. 1996;49(1):1–49.

[5] Maynard DC, Joseph TA, Maynard AM. Underemployment, job attitudes, and turnover intentions. J Organ Behav. 2006;27(4):509–36.

[6] Debus ME, Körner B, Wang M, Kleinmann M. Reacting to Perceived Overqualification: Uniting Strain-Based and Self-Regulatory Adjustment Reactions and the Moderating Role of Formal Work Arrangements. J Bus Psychol [Internet]. 2023;38(2):411–35. Available from: 10.1007/s10869-022-09870-8 10.1007/s10869-022-09870-8 PMC9852809 36694852

[7] Neto JMG, Borges-Andrade JE. Perceived overqualification and job attitudes of public servants. Rev Bras Gest Negocios. 2024;26(1).

[8] Guo YF, Fan JY, Lam L, Plummer V, Cross W, Ma YZ, et al. Associations between perceived overqualification, transformational leadership and burnout in nurses from intensive care units: A multicentre survey. J Nurs Manag. 2022;30(7):3330–9. 36042016 10.1111/jonm.13774

[9] Klieb SFG, Elghabbour GMM, Hassane SMA, El-Said HDA. Influence of perceived ethical work climate on workplace alienation among nursing staff at minia university hospital. Int Egypt J Nurs Sci Res. 2023;3(2):726–41.

[10] Bakker AB, Demerouti E. The job Demands-Resources model: state of the Art. J Manag Psychol. 2007;22(3):309–28.

[11] Uddin MK, Azim MT, Islam MR. Effect of perceived overqualification on work performance: influence of moderator and mediator. Asia Pac Manag Rev. 2023;28(3):276–86.

[12] Weinberg I, Ronningstam E. Narcissistic personality disorder: progress in Understanding and treatment. Focus (Madison). 2022;20(4):368–77. 10.1176/appi.focus.20220052 PMC10187400 37200887

[13] O’Reilly CA, Hall N. Grandiose narcissists and decision making: impulsive, overconfident, and skeptical of experts–but seldom in doubt. Pers Individ Dif. 2020;168:1–12. 10.1016/j.paid.2020.110280 PMC7427600 32834287

[14] Moon C, Morais C. The effect of covert narcissism on workplace incivility: The mediating role of self-esteem and norms for respect. Curr Psychol [Internet]. 2023;42(21):18108–22. Available from: 10.1007/s12144-022-02968-5

[15] Canbolat MA, Bedük A. The mediating role of perceived overqualification in the effect of the dark triad on organizational cynicism. Selçuk Üniversitesi Sos Bilim Enstitüsü Derg. 2023;(51):391–407.

[16] Ahmad J, Zahid S, Wahid FF, Ali S. Impact of role conflict and role ambiguity on job satisfaction the mediating effect of job stress and moderating effect of Islamic work ethics. Eur J Bus Manag Res. 2021;6(4):41–50.

[17] Zhang H li, Wu C, Yan J, ran, Liu J hua, Wang P, Hu M, yi et al. The relationship between role ambiguity, emotional exhaustion and work alienation among chinese nurses two years after COVID-19 pandemic: a cross-sectional study. BMC Psychiatry. 2023;23(1):1–9. 10.1186/s12888-023-04923-5 PMC10355025 37464335

[18] Wiegand JP. When overqualification turns dark: A moderated-mediation model of perceived overqualification, narcissism, frustration, and counterproductive work behavior. Pers Individ Dif [Internet]. 2023;214(August):112351. Available from: 10.1016/j.paid.2023.112351

[19] Higgins ET. Self-Discrepancy theory: what patterns of Self-Beliefs cause people to suffer?? Adv Exp Soc Psychol. 1989;22(C):93–136.

[20] Chaudhury A, Mallick D. Can an ego defense mechanism model help explain dysfunctional is security behavior? In: Americas Conference on Information Systems. 2018: Digital Disruption, AMCIS 2018. 2018.

[21] Khan AN, Soomro MA, Khan NA, Bodla AA. Psychological dynamics of overqualification: career anxiety and decision commitment in STEM. BMC Psychol [Internet]. 2024;12(1). Available from: 10.1186/s40359-024-02061-5 10.1186/s40359-024-02061-5 PMC11583426 39578864

[22] Tang H, Liu Y, Loi R, Chow CWC, Jiang N. Role ambiguity and work alienation during the COVID-19 pandemic: the perspective of occupational disidentification. J Manag Psychol. 2024;39(2):117–30.

[23] Mwakyusa JRP, Mcharo EW. Role ambiguity and role conflict effects on employees’ emotional exhaustion in healthcare services in Tanzania. Cogent Bus Manag [Internet]. 2024;11(1). Available from: 10.1080/23311975.2024.2326237

[24] Maynard DC, Joseph TA, Maynard AM. Underemployment, job attitudes, and turnover intentions. J Organ Behav. 2006;27:509–36.

[25] Lee HM, Chou MJ, Wu HT. The measurement of perceived overqualification and the relationships among perceived overqualification, psychological empowerment, job satisfaction of private kindergarten teachers. Eur J Res Soc Sci. 2016;4(8):1–15.

[26] Mottaz CJ. Some determinants of work alienation**. Sociol Q. 1981;22(4):515–29.

[27] AbdEl-Monem AM, Zaki AKAEA, Hasanin AG. Organizational Cynicism and Work Alienation among Nurses and Its Relation to Organizational Loyalty. Assiut Sci Nurs J [Internet]. 2023;11(38):227–37. Available from: https://asnj.journals.ekb.eg/article_320460.html%0Aasnj.journals.ekb.eg/article_320460_502a8e2477cb3e90d8c43fbd87ca94f3.pdf

[28] Mohamed LK, Abou Shaheen RAEM. Influence of perceived organizational injustice on workplace alienation among nursing staff during COVID-19 pandemic. Int Egypt J Nurs Sci Res. 2022;2(2):362–77.

[29] Rizzo JR, House RJ, Lirtzman SI. Role conflict and ambiguity in complex organizations. Adm Sci Q. 1970;15(2):150–63.

[30] Smith CS, Tisak J, Schmieder RA. The measurement properties of the role conflict and role ambiguity scales: A review and extension of the empirical research. J Organ Behav. 1993;14(1):37–48.

[31] Alyahya SA, Al-Mansour KA, Alkohaiz MA, Almalki MA. Association between role conflict and ambiguity and stress among nurses in primary health care centers in Saudi Arabia during the coronavirus disease 2019 pandemic A cross-sectional study. Med (United States). 2021;100(37):1–5. 10.1097/MD.0000000000027294 PMC8447988 34664892

[32] Furukawa R, Driessnack M, Colclough Y. A committee approach maintaining cultural originality in translation. Appl Nurs Res [Internet]. 2014;27(2):144–6. Available from: 10.1016/j.apnr.2013.11.011 10.1016/j.apnr.2013.11.011 24332480

[33] Brislin RW. Back-translation for cross-cultural research. J Cross-Cult Psychol. 1970;1:185–216.

[34] Matherne CF, Credo KR, Gresch EB, Lanier PA. Exploring the relationship between Covert narcissism and amorality: the mediating influences of Self-efficacy and psychological entitlement. Am J Manag. 2019;19(5):31–9.

[35] Khan J, Zhang Q, Saeed I, Ali A, Fayaz M. Unveiling the nexus between perceived overqualification and knowledge hiding: Moderated mediation analysis of job crafting and job boredom. Heliyon [Internet]. 2024;10(10):e31701. Available from: 10.1016/j.heliyon.2024.e31701 10.1016/j.heliyon.2024.e31701 PMC11145549 38831809

[36] Fossati A, Borroni S, Eisenberg N, Maffei C. Relations of proactive and reactive dimensions of aggression to overt and Covert narcissism in nonclinical adolescents. Aggress Behav. 2010;36(1):21–7. 19918915 10.1002/ab.20332 PMC2805785

[37] Di Pierro R, Mattavelli S, Gallucci M. Narcissistic traits and explicit self-esteem: the moderating role of implicit self-view. Front Psychol. 2016;7(NOV):1–9. 27920739 10.3389/fpsyg.2016.01815 PMC5118622

[38] Grapsas S, Brummelman E, Back MD, Denissen JJA. The why and how of narcissism: A process model of narcissistic status pursuit. Perspect Psychol Sci. 2020;15(1):150–72. 31805811 10.1177/1745691619873350 PMC6970445

[39] AL-Ghwary AA, AL-Oweidat IA, Al-Qudimat AR, Abu Shosha GM, Khalifeh AH, ALBashtawy M. The impact of work environment on structural empowerment among nurses in governmental hospitals. Nurs Rep. 2024;14(1):482–93. 38535709 10.3390/nursrep14010037 PMC10975519

[40] Khassawneh O, Mohammad T, Momany MT. Perceived overqualification and job outcomes: the moderating role of manager envy. Sustain. 2023;15(1):1–18.

[41] Guo YF, Wang Y, Plummer V, Cross W, Lam L, Wang K. fang. Associations between perceived overqualification, organisational commitment and work passion of nurses: A multicentre cross-sectional study. J Nurs Manag. 2022;30(5):1273–82. 10.1111/jonm.13610 35338533

[42] Cengiz A, Yoder LH, Danesh V. A concept analysis of role ambiguity experienced by hospital nurses providing bedside nursing care. Nurs Heal Sci. 2021;23(4):807–17. 10.1111/nhs.12888 34689398

[43] Zhu C, Su R, Zhang X, Liu Y. Relation between narcissism and meaning in life: the role of conspicuous consumption. Heliyon [Internet]. 2021;7(9):e07885. Available from: 10.1016/j.heliyon.2021.e07885 10.1016/j.heliyon.2021.e07885 PMC8426529 34522799

[44] Judeh M. Role ambiguity and role conflict as mediators of the relationship between orientation and organizational commitment. Int Bus Res. 2011;4(3):171–81.

[45] Unguren E, Arslan S. The effect of role ambiguity and role conflict on job performance in the hotel industry: the mediating effect of job satisfaction. Tour Manag Stud. 2021;17(1):45–58.

